# Laboratory indices of hospitalized sickle cell disease patients, prevalence and antimicrobial susceptibility of pathogenic bacterial isolates at MRCG ward in the Gambia

**DOI:** 10.1186/s12879-023-08542-z

**Published:** 2023-08-22

**Authors:** Mustapha Dibbasey, Mamudou Dahaba, Francess Sarfo, Ida Jallow-Manneh, Buntung Ceesay, Solomon Umukoro, Mouhamadou Fadel Diop, Alfred Amambua-Ngwa

**Affiliations:** 1 Department of Haematology laboratory, Clinical Laboratory, Medical Research Council the Gambia at London School of Hygiene and Tropical Medicine, Fajara, The Gambia; 2 Department of Clinical Microbiology laboratory, Clinical Laboratory, Medical Research Council the Gambia at London School of Hygiene and Tropical Medicine, Fajara, The Gambia; 3 Clinical Laboratory Department, Medical Research Council the Gambia at London School of Hygiene and Tropical Medicine, Fajara, The Gambia; 4 Department of Data Science, Medical Research Council the Gambia at London School of Hygiene and Tropical Medicine, Fajara, The Gambia; 5 Malaria Biology Group Department, Medical Research Council the Gambia at London School of Hygiene and Tropical Medicine, Fajara, The Gambia

**Keywords:** Sickle cell disease, Bacteraemia, Bacterial infections, Antimicrobial resistance patterns, Haematological parameters

## Abstract

**Background:**

The aim of this study was to determine the prevalence of invasive bacterial infections and their antimicrobial resistance patterns in sickle cell disease (SCD) patients admitted at the Medical Research Council the Gambia (MRCG) Ward in the era of PCV and Hib vaccination in the Gambia.

**Methods and Results:**

This study was conducted in the clinical laboratory department of MRCG. We retrospectively generated haematological, and blood culture data from our electronic medical records from 2015 to 2022 of SCD patients admitted to MRCG Ward. Of 380 SCD patients, blood culture was requested only for 159. Of the 159 admitted SCD, 11 patients had qualified positive blood cultures. Five different types of bacterial pathogens were isolated from these positive blood cultures: 4 *Staphylococcus aureus*, 3 *Streptococcus pneumoniae*, 2 Salmonella species, 1 Enterococcus species, and 1 *Shigella boydii*. No episode of bacteremia caused by *Haemophilus influenzae* type b was identified. The molecular serotyping of the *Streptococcus pneumoniae* isolates revealed non-vaccine serotypes 10 A, 12 F and 12 F. Penicillin resistance was recorded in two of the three *Streptococcus pneumoniae*. The *Staphylococcus aureus* isolates were penicillin resistant but cefoxitin sensitive, hence no methicillin (oxacillin) resistant Staphylococcus aureus was reported. Generally, the isolated pathogens were all sensitive to chloramphenicol, and vancomycin. The haematological indices were not significantly varied between SCD patients with and without microbiologically confirmed bacterial infection.

**Conclusion:**

*Streptococcus pneumoniae* and *Staphylococcus aureus* were the most common cause of bacteremia in these admitted SCD patients. The presence of non-typhoidal Salmonella and Shigella infection coupled with penicillin resistance should be considered during penicillin prophylaxis and empirical treatment regimens for SCD patients and future SCD management policies in the Gambia. The haematological parameters may not be reliable biomarkers in differentiating bacterial from non-bacterial infections in SCD patients.

**Supplementary Information:**

The online version contains supplementary material available at 10.1186/s12879-023-08542-z.

## Introduction

Sickle cell disease (SCD) is recognised as a major global health problem with over 70% of children born with SCD being from Africa where medical care and public health interventions are suboptimal [1–3]. Cross-sectional studies conducted in rural Gambia have estimated the SCD prevalence to be around 0.8–1.2% in the newborn and sickle cell trait to be around 15–20% [4–6] .

SCD patients are predisposed to several SCD related complications such as vasocclusive crisis, asplenia, priapism, stunted growth, a haematological crisis such as severe anaemia, and bacterial infection. Despite several other complications encountered by SCD patients during the first few years of life, invasive bacterial infection is the leading cause of morbidities and mortalities in SCD patients [7–11]. This is because most SCD patients develop functional asplenism in their early years of life because of repeated episodes of sickling-induced splenic infarction which predisposes them to episodes of infections caused by encapsulated bacteria such as such as *Streptococcus Pneumoniae, Haemophilus influenzae type b*, and non-typhoidal *Salmonella species* [22–24].

The two major prophylactic interventions are vaccination, and daily oral penicillin at least until the fifth year [12, 13]. Despite the limited accessibility to vaccinations and routine penicillin prophylaxis in most health facilities in sub-Saharan Africa, the Gambia has a well robust vaccination programme with the introduction of a 7-valent pneumococcal conjugate vaccine (PCV) in 2009 and then 13-valent PCV in 2011, and *Haemophilus influenzae type b* (Hib) vaccine in 1997 in Expanded Program on Immunization (EPI). The introduction of PCV and Hib vaccine into EPI in the Gambia has drastically reduced the bacterial burden in the Gambia [14, 15]. A retrospective study by Soothill et al. (2016) spanning 2010 to 2015 in the Gambia revealed no episodes of *Streptococcus pneumoniae* and Hib infection among SCD patients hospitalised at the Medical Research Council the Gambia (MRCG) clinic Ward. However, no data was reported on the antimicrobial resistance pattern of other bacterial pathogens recorded in Soothill et al. (2016) study, despite global reports of widespread antimicrobial resistance [16, 17]. The results of a recent systematic review show that antimicrobial resistance caused around 1.29 million deaths in 2019, and antimicrobial-resistant infections contributed to around 4.95 million deaths globally [17].

The lack of rapid diagnostic microbiological tools means that many SCD patients with febrile illness are treated empirically with penicillin. Consequently, this is contributing to the emergence of penicillin-resistant and multidrug-resistant pathogenic strains in many parts of the World and becoming widespread in some less developed communities [16]. Therefore, knowledge of the most common pathogens infecting patients with SCD, and their antimicrobial resistance patterns could be used to improve antimicrobial prophylaxis and empirical treatments for bacterial infections for SCD patients in the Gambia. The aim of this study was to provide an insight into the burden of invasive bacterial infections among SCD patients hospitalised at the Medical Research Council the Gambia (MRCG) clinic Ward in the era of PCV and Hib vaccine post-introduction in the Gambia EPI. Considering widespread antimicrobial resistance in the global context, we also aim was to probe the antimicrobial resistance patterns of detected pathogens to guide appropriate antibiotic prophylaxis and empirical treatment for SCD patients and haematological indices distribution in SCD population with or without microbiologically confirmed bacterial infection in the blood.

## Methods

### Study setting

The study was conducted in clinical laboratory department hosted at the Kuyateh Building at MRCG@LSHTM. The clinical laboratory of MRCG is in the Kanifing municipality, greater Banjul area, the Gambia. The Gambia is geographically situated in West Africa with a population of about 2.5 million and an area of 10,000km^2^. SCD and infectious diseases are prevalent in the Gambia and SCD patients are given penicillin as prophylaxis [14, 15].

Clinical laboratory departments composed of clinical microbiology, clinical haematology and clinical biochemistry laboratories attained good clinical laboratory practice in 2011 [18] and ISO15189 accreditation in 2016 [19, 20] with laboratory and clinical data captured and managed through a certified electronic medical records system. The responsibility of the clinical laboratory department is to provide timely and accurate diagnosis of various diseases including SCD. The laboratories provide services to clinical services at MRCG, clinical trial projects, as well as government and private hospitals. Annually, the haematology laboratory analyses more than two thousand genotype samples.

### Study design, population, data collection

This study was a retrospective study targeting SCD patients with blood culture requests that were admitted to the MRCG clinical Ward over a period of 7 years, from January 2015 to July 2022. The blood cultures were drawn from hospitalized patients with suspected, confirmed or self-reported SCD with clinical features suggestive of infections (e.g. fever). 

**Fig. 1:**
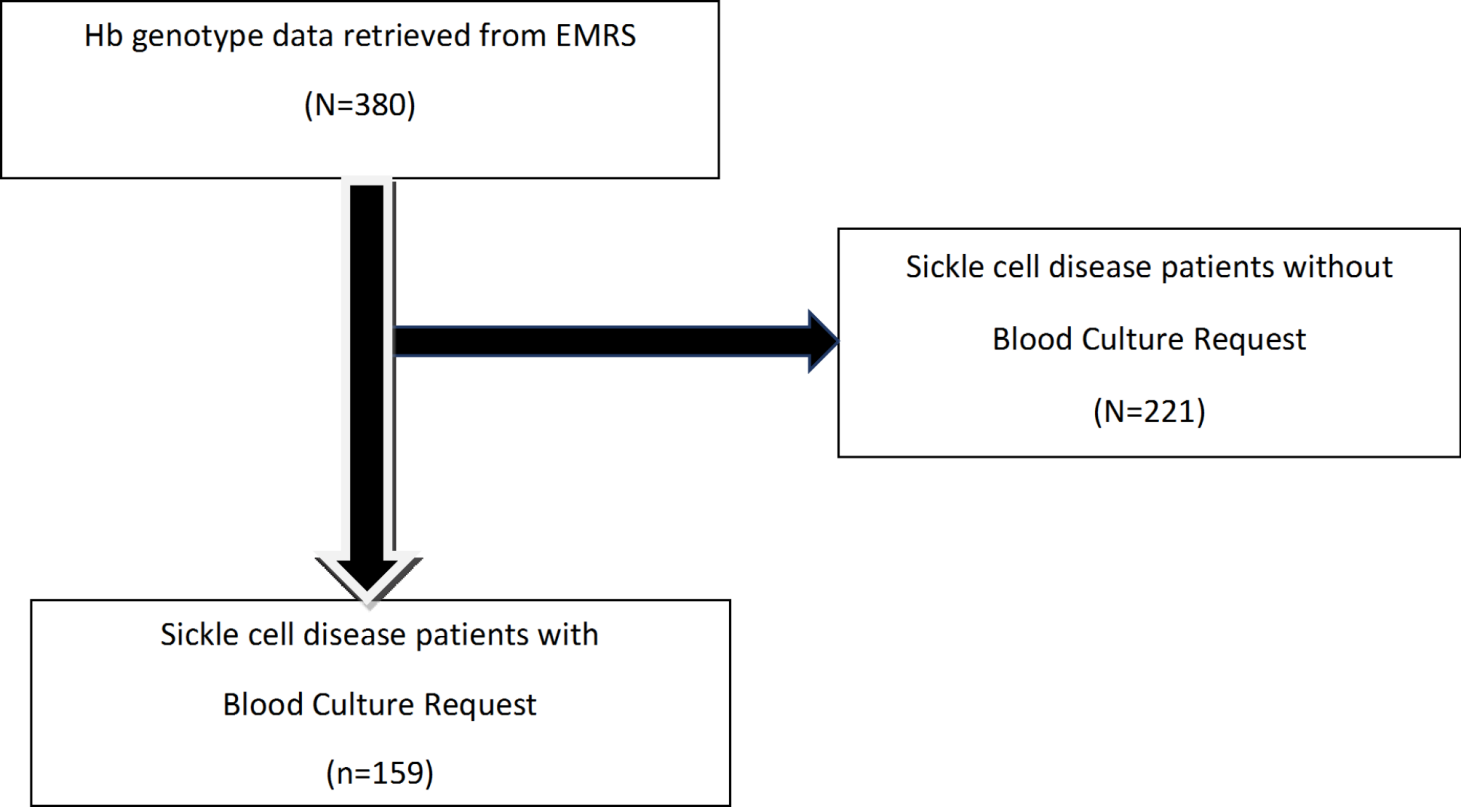
Flow chart demonstrating the steps that were taken to select 159 SCD patients for analysis

Data from 159 SCD patients with blood culture results were retrieved from the electronic medical records system as shown in Fig. 1. The following laboratory measurements were extracted: full blood count results, haemoglobin, red blood count, mean cell volume, white cell count and platelet count. In addition to their blood culture results, the data for antimicrobial susceptibility test was also retrieved for bacterial pathogens that were isolated.

### Laboratory method

SCD was diagnosed in the haematology laboratory by alkaline-based haemoglobin electrophoresis method (Helena Laboratories). All the 159 SCD patients had full blood count results determined either with 3-part Medonic M-series analyzer (Boule, Sweden), 5-part HumaCount 5D analyzer (Human, Germany), 5-part Cell Dyn Ruby analyzer (Abbott Diagnostics, USA) or 5-part Sysmex XN-1500 haematology analyzer (Sysmex, Germany).

For blood culture requests for SCD in-patients, aerobic and anaerobic blood culture bottles were collected and sent to the microbiology laboratory for incubation in BD Bactec™ 9050 Blood Culture System (Becton Dickinson and Company, UK). Positive blood culture bottles were retrieved from the BD Bactec™ 9050 Blood Culture System and cultured for bacterial growth. No further microbiological procedures were performed on samples with reported contaminants. However, blood cultures with qualified bacterial growth underwent a series of biochemical tests such as the Analytical Profile Index, followed by antibiotic susceptibility testing. Antibiotic susceptibility testing was performed using Kirby-Bauer disc diffusion method following isolation of bacterial pathogens. The interpretation of antimicrobial susceptibility was done by measuring the diameter of the zone of inhibition and comparing it to predefined values provided by 2017 Clinical Laboratory Standard Institute (CLSI) guidelines [54].

### Molecular serotyping

As per microbiological procedure, the *Streptococcus pneumonia* isolates detected in the SCD patients were subcultured in the microbiology laboratory and DNA extraction was performed using a Qiagen DNA extraction kit (QIAamp Genomic DNA kit; Qiagen) in the Molecular laboratory. The isolates were serotyped using sequential multiplex polymerase chain reaction in the Molecular Microbiology Laboratory as previously described [21].

### Statistical method

The data was analyzed using SPSS package (v2) where a frequency table on ordinal and nominal variables was generated for the number of pathogens isolated (positive blood culture), blood cultures without bacterial growth (No bacterial growth), and contaminated blood cultures. R package was used to display the antibiotic susceptibility patterns of all the 11 pathogens isolated against a spectrum of antibiotics. With a relatively small number of bacterial infections detected, a pairwise matched infected versus non-infected SCD analysis was performed.

## Results

From January 2015 to July 2022, blood culture samples were collected from 159 SCD patients; females accounting for 49.1% (78/159) and males for 50.9% (81/159) (Tables 1 and 2). Of the 159 SCD patient blood culture requests, 138 (86.8%) yielded no bacterial growth whilst 10 (6.3%) grew contaminants that were coagulase-negative *Staphylococcus* and *Bacillus species*. Eleven blood cultures grew pathogenic micro-organisms as shown in Table 1 accounting for 7% of blood culture samples received in the clinical microbiology laboratory. From the 11 positive blood cultures, 5 different types of pathogens were isolated which comprised *Staphylococcus aureus* (4/11), *Streptococcus pneumoniae* (3/11), *Salmonella species* (2/11), *Shigella boydii* (1/11), and *Enterococcus species* (1/11) (Table 1). No episode of bacteremia caused by *Haemophilus influenza* type b was identified and one SCD patient was serologed positive for hepatitis B virus. Blood cultures with contaminants and the patient with hepatitis B were categorised under the group of “No bacterial growth” as shown in Table 1. To further characterize the *Streptococcus Pneumoniae* isolates, molecular serotyping was performed. The molecular serotyping of the *Streptococcus pneumoniae* isolates revealed serotypes 10 A, 12 F and 12 F as shown in Table 3.

**Table 1:** Frequency of blood culture samples and pathogens isolated from positive blood cultures with bacterial growth in admitted patients with SCD

Characteristics	Frequency	Percentage (%)
**Blood Culture Requested**		
No bacterial growth	138	86.8
Bacterial growth	11	6.9
Contaminants	10	6.3
Total	159	100.0
**Pathogens isolated**		
Enterococcus species	1	9.1
Salmonella species	2	18.2
Shigella boydii	1	9.1
Staphylococcus aureus	4	36.2
Streptococcus pneumonia	3	27.3
Total	11	100

**Table 2:** Characteristics of SCD patients including their SCD status

Parameters	SCD patients (n = 159)	Bacterial Growth (n = 11)	No Bacterial Growth (n = 147)
Gender (n, %)			
Female	78(49.1)	4(36.4)	74(50.3)
Male	81(50.9)	7(63.6)	73(49.7)
Age (n, %)			
0-4years	66(41.77)	8(72.73)	58(39.46)
5-9years	41(25.95)	1(9.09)	40(27.21)
10-14years	15(9.49)	1(9.09)	14(9.52)
>=15	36 (22.78)	1(9.09)	35(23.81)
SCD Status			
HbSS	154(96.86)	9(81.82)	144(97.96)
HbSC	5(3.14)	2(18.18)	3(2.04)

**Table 3:** Serotypes of *Streptococcus pneumoniae* isolated from SCD patients

Patient ID	Age (yr)	*Streptococcus Pneumoniae* Serotype	PCV-13 Vaccine Serotype (YES/NO)
2020/0167	0	10A	NO
2003/A804	32	12 F	NO
2021/2426	1	12 F	NO

**Fig. 2:**
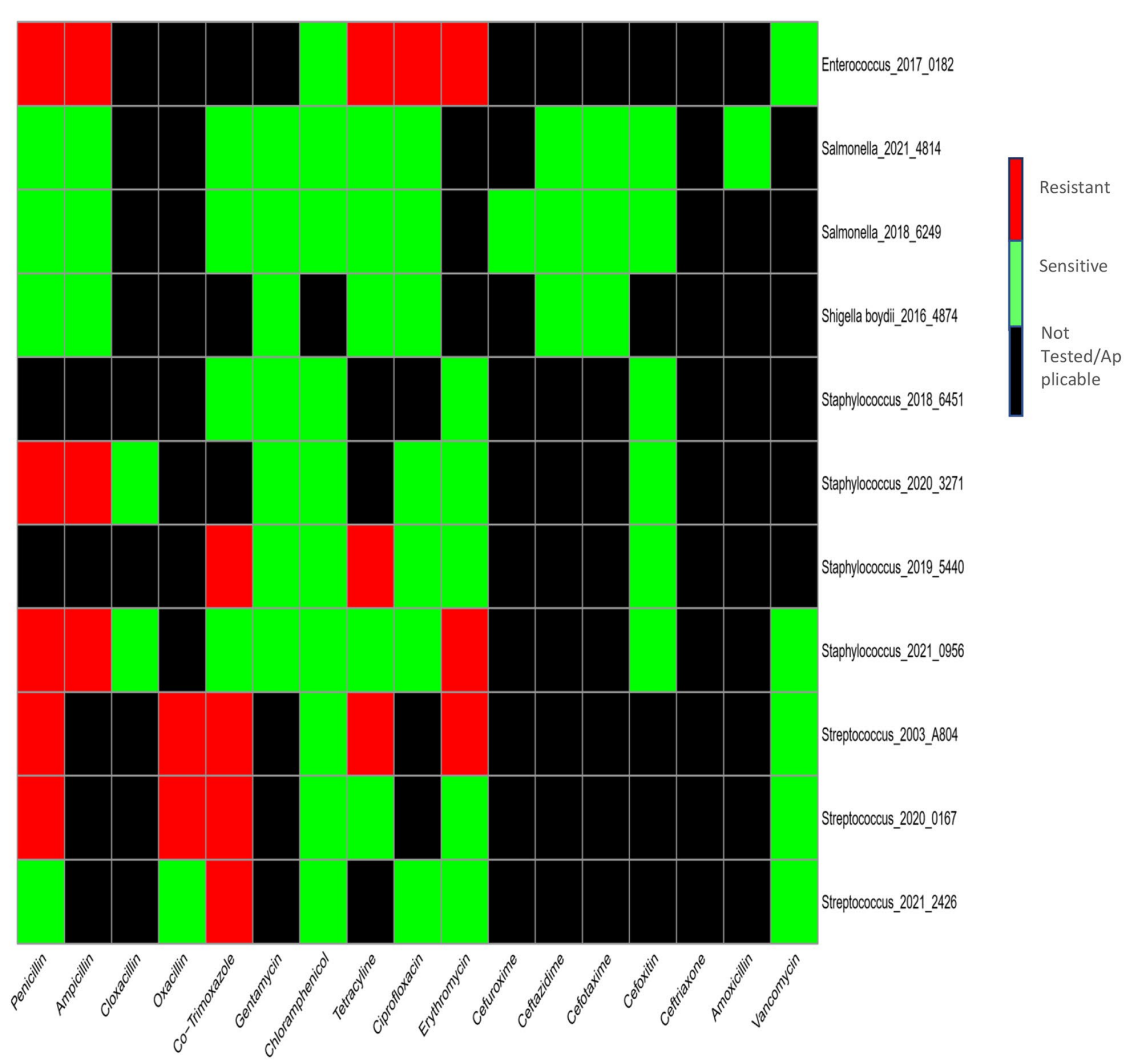
Graphical display of AST results of pathogens isolated from positive blood culture samples of SCD patients. Not available/applicable means the antibiotic was not tested against or applicable for bacterial pathogen isolated from the positive blood culture

Antimicrobial susceptibility tests were performed on all the pathogens isolated as shown in Fig. 2 using 2017 CLSI guidelines ^(54)^. All three *Streptococcus pneumoniae isolates* detected were resistant to cotrimoxazole whereas two of the three *Streptococcus pneumoniae isolates* were resistant to penicillin. The *Streptococcus pneumoniae* isolates were susceptible to chloramphenicol, and vancomycin. One of the three *Streptococcus pneumoniae* isolates showed resistance to erythromycin. An incomplete antibiotic susceptibility testing (AST) data for *Streptococcus pneumoniae* was found for tetracycline as only two of the three isolates were tested for tetracycline and no AST data for cefuroxime.

All the four *Staphylococcus aureus pathogens* isolated in the blood culture of SCD patients were resistant to penicillin but susceptible to gentamycin, chloramphenicol, ciprofloxacin and cefoxitin (which is currently tested as a surrogate for oxacillin). Based on cefoxitin AST result, no methicillin (cefoxitin) resistant *Staphylococcus aureus* was isolated in SCD patients. Salmonella and Shigella isolates were susceptible to all the antibiotics they were tested against, hence 100% susceptibility as shown in Fig. 2.

The patients characteristics and hematological indices of the 159 SCD patients are summarized in Tables 2 and 4 and supplementary Table 1. Comparing haematological values between patients with bacterial and non-bacterial growth, no statistically significant difference (P > 0.05) was found in all the five main full blood count (FBC) parameters as shown in Table 4.

**Table 4:** Haematological indices of SCD patients with bacterial growth and without bacterial growth using pairwise mean comparison

Main FBC Parameters		SCD patients (n = 159)	Bacterial Growth (n = 11)	No Bacterial Growth (n = 11)	P value
HB (g/dL) (mean)		7.82	8.58_a_	7.30_a_	P > 0.05
WBC (103/uL) (mean)		18.26	18.51_a_	21.66_a_	P > 0.05
RBC (106/uL) (mean)		2.91	3.34_a_	2.86_a_	P > 0.05
MCV (fL) (mean)		81.3	79.4_a_	76.2_a_	P > 0.05
PLATS (103/uL) (mean)		442.0	478_a_	400_a_	P > 0.05

Note: Values in the same row and subtable sharing the same subscript are **NOT** significantly different at p< 0.05 in the two−sided test of equality for column means

## Discussion

Bacterial infection is one of the major causes of morbidities and mortalities of SCD in sub-Saharan Africa. From our retrospective data, 5 different types of bacterial pathogens were isolated: *Staphylococcus aureus* (36.2%), *Streptococcus pneumoniae* (27.3%), *Salmonella species* (18.2%), *Shigella boydii* (9.1%), and *Enterococcus species* (9.1%). The data has shown pathogens isolated were mainly encapsulated bacteria except for *Staphylococcus aureus* as shown in other studies [6, 7, 11].

Our retrospective study has revealed 7 positive blood cultures from 159 blood culture requested for SCD patients hospitalised at MRCG clinic Ward from 2015 to 2022. Our result is comparable to the results reported in two retrospective studies conducted in the Gambia (8.4%) and Cameroon (9.7%), and a case-control study result conducted in Kenya (6%) [6, 7, 11]. Despite the massive immunisation program in the Gambia that has integrally incorporated PCV7 and PCV13, bacterial infection remains one of the major causes of morbidity and mortality in the Gambian SCD patients. Thus, efforts to promote immunisation and antibiotic prophylactic interventions for SCD patients at an early age should be intensified [14, 15, 25].

*Streptococcus pneumoniae* was isolated in three patients among the 11 cases of bacterial infections (3/11) in SCD patients. This differs from Soothill et al. 2016 study result that reported no episodes of *Streptococcus pneumoniae* in SCD patients from 2010 to 2015 in the Gambia. Two of the three *Streptococcus pneumonia* were isolated from SCD patients who were below 5 years of age. Further molecular serotyping of the isolates revealed non-vaccine *Streptococcus pneumoniae* serotypes (10 A, 12 F and 12 F). This indicates a plausible emergence of non-vaccine serotypes in the Gambia, consistent with previous studies conducted in the Gambia by Roca et al. 2016 and Mackenzie et al. 2021 as well as reports from other countries following the introduction of 7-valent and 13-valent PCV [26–33].The emergence of non-vaccine serotypes is worrying and may be associated with heightened antimicrobial resistance due to selective pressure, and virulence [33]. It is important to put in place continued pneumococcal serotype surveillance mechanisms for the emergence of non-vaccine serotypes. The incorporation of 20-valent PCV with expanded serotypes coverage including serotypes 10 A and 12 F and improved immunogenicity in EPI will be critical for optimal prevention of invasive pneumococcal disease in SCD patients [28, 34].

Understanding the antimicrobial resistance pattern is critically important in the management of SCD patients, particularly in the direction of prophylaxis and empirical treatment. Except for *Salmonella* and *Shigella* isolates, almost all the bacterial pathogens (*Streptococcus pneumoniae*, *Staphylococcus aureus* and *Enterococcus species*) isolated in this study were resistant to penicillin and this is likely due to widely use penicillin antibiotic as prophylaxis for SCD patients in both primary healthcare centres and tertiary hospitals in the Gambia [35, 36]. Also, the finding showed all three *Streptococcus pneumoniae* isolates were resistant to co-trimoxazole which is in line with a study conducted in Ghana showing an extremely high level of cotrimoxazole resistance in *Streptococcus pneumoniae* [37]. Cotrimoxazole is widely used at primary healthcare facilities in the Gambia which could have contributed to this high level of resistance. However, no resistance was reported against vancomycin and chloramphenicol for all the 11 pathogens isolated. Unlike vancomycin, chloramphenicol is not recommended for routine use due to the considerable side effects associated with this antibiotic [38, 39].

Generally, our data showed low haemoglobin 9(g/dL) and RBC (10^6^/uL) values similar to several previous studies [40–44]. Chronic haemolytic anaemia is one of the prominent pathological features of SCD that accompanies an increased tendency of RBC lysis and adhesion which shortens RBC lifespan. Persistent chronic haemolytic anaemia in SCD characterised by ongoing RBC lysis and splenic sequestration could explain this overall reduction of red blood cell count and haemoglobin in both bacterial infected and nonbacterial infected SCD patients [44–47]. It was not surprising that the WBC count was generally elevated in SCD patients regardless of the infection status, with very high WBC counts (18.26 × 10^3^/uL) in both bacterial and nonbacterial infected SCD patients and this is consistent with previous studies [48–50]. Even though several studies attributed elevated WBC count to bacterial infections in SCD patients, the mean WBC count in bacterial infected SCD patients (18.51 × 10^3^/uL) was not significantly different from the mean WBC count in nonbacterial infected SCD patients’ population (21.66 × 10^3^/uL) in this study which could be attributed to systemic chronic inflammation**.** Elevated WBC count predisposes SCD patients to severe SCD crisis and serves as a risk factor for early SCD-related mortality, stroke, and acute chest syndrome [51–53].

The study has significant limitations which could be addressed by a well-designed prospective study on the SCD population congruent to the case-control study approach conducted in Kenya by Williams et al. 2009. The previous vaccination record of SCD patients was not available in the electronic medical record system database. Hence, we could not confirm whether the *Streptococcus pneumoniae* isolates were isolated from SCD patients who were fully vaccinated with PCV13 even though the serotypes (10 A and 12 F) were non-vaccine serotypes [34]. Our data on AST is slightly inadequate, hence it was difficult to decipher antimicrobial resistance pattern in the data. For instance, tetracycline was tested against two of the three *Streptococcus pneumoniae* isolated as well as two of the four *Staphylococcus aureus* isolates.

## Conclusion

*Streptococcus pneumoniae* and *Staphylococcus aureus* accounted for the most common cause of bacteremia in this SCD patients. Penicillin antimicrobial resistance was generally high. Thus, the presence of non-typhoidal *Salmonella* and *Shigella* infections coupled with high penicillin antimicrobial resistance should be considered when prescribing prophylaxis and empirical treatment regimens for SCD patients, particularly in the Gambia. The haematological parameters may not be reliable biomarkers in differentiating bacterial from non-bacterial infections in SCD patients.

### Electronic supplementary material

Below is the link to the electronic supplementary material.

Supplementary Material 1

Supplementary Material 2

## Data Availability

The datasets used and/or analysed during the current study are included in this published article and its supplementary information files.

## Abbreviations

SCD: Sickle cell disease
PCV: Pneumococcal Conjugate vaccine
MRCG: Medical Research Council the Gambia
LSHTM: London School of Hygiene and Tropical Medicine
MRCG@LSHTM: Medical Research Council the Gambia at London School of Hygiene and Tropical Medicine
ISO15189: International Standard for medical laboratories
SPSS: Statistical Package for Social Science
WBC: White blood cells
RBC: Red Blood Cells
HiB: Haemophilus influenzae type b
HB: Haemoglobin

## References

[1] WHO. Sickle-cell anaemia report. World Health. 2006.

[2] Piel FB, Hay SI, Gupta S, Weatherall DJ, Williams TN. Global burden of Sickle Cell Anaemia in Children under five, 2010–2050: Modelling based on demographics, excess mortality, and interventions. PLoS Med. 2013;10(7). 10.1371/journal.pmed.1001484 PMC3712914 23874164

[3] Wonkam A, Makani J. Sickle cell disease in Africa: an urgent need for longitudinal cohort studies. Lancet Glob Heal [Internet]. 2019;7(10):e1310–1. Available from: 10.1016/S2214-109X(19)30364-X. 10.1016/S2214-109X(19)30364-X PMC7255820 31451442

[4] Allen SJ, Bennett S, Riley EM, Rowe PA, Jakobsen PH, O’Donnell A et al. Morbidity from malaria and immune responses to defined Plasmodium falciparum antigens in children with sickle cell trait in The Gambia. Trans R Soc Trop Med Hyg [Internet]. 1992;86(5):494–8. Available from: 10.1016/0035-9203(92)90083-O. 10.1016/0035-9203(92)90083-o 1475814

[5] Cox1 SE, Doherty2 CP, Sarah H et al. Atkinson1, 2 CVN, Fulford1 AJC, Sirugo3 G, Rockett4 KA,. Haptoglobin genotype, anaemia and malaria in Gambian children. Trop Med Int Heal [Internet]. 2008;13(1):76–82. Available from: 10.1111/j.1365-3156.2007.01976.x. 10.1111/j.1365-3156.2007.01976.x 18291005

[6] Soothill G, Darboe S, Bah G, Bolarinde L, Cunnington A, Anderson ST (2016). Invasive bacterial infections in gambians with sickle cell anemia in an era of widespread pneumococcal and hemophilus influenzae type b vaccination. Med (Baltim).

[7] Williams TN, Uyoga S, Macharia A, Ndila C, McAuley CF, Opi DH et al. Bacteraemia in Kenyan children with sickle-cell anaemia: a retrospective cohort and case-control study. Lancet [Internet]. 2009;374(9698):1364–70. Available from: 10.1016/S0140-6736(09)61374-X. 10.1016/S0140-6736(09)61374-X PMC2768782 19747721

[8] Williams TN. Sickle cell disease in Sub-Saharan Africa. Hematol Oncol Clin N Am. 2016; Vol. 30. 10.1016/j.hoc.2015.11.005 PMC6858853 27040958

[9] Novelli EM, Gladwin MT. Crises in sickle cell disease. Chest [Internet]. 2016;149(4):1082–93. Available from: 10.1016/j.chest.2015.12.016. 10.1016/j.chest.2015.12.016 PMC6637264 26836899

[10] Meremikwu MM, Okomo U. Sickle cell disease. BMJ Clin Evid. 2011:1–19. PMC3217656 21718552

[11] Alima Yanda AN, Nansseu JRN, Mbassi Awa HD, Tatah SA, Seungue J, Eposse C (2017). Burden and spectrum of bacterial infections among sickle cell disease children living in Cameroon. BMC Infect Dis.

[12] Okuonghae HO, Nwankwo MU, Offor EC (1993). Pattern of bacteraemia in febrile children with sickle cell anaemia. Ann Trop Paediatr.

[13] Akuse RM (1996). Variation in the pattern of bacterial infection in patients with sickle cell disease requiring admission. J Trop Pediatr.

[14] Mackenzie GA, Hill PC, Jeffries DJ, Hossain I, Uchendu U, Ameh D (2016). Effect of the introduction of pneumococcal conjugate vaccination on invasive pneumococcal disease in the Gambia: a population-based surveillance study. Lancet Infect Dis.

[15] Mackenzie GA, Hill PC, Jeffries DJ, Ndiaye M, Sahito SM, Hossain I et al. Impact of the introduction of pneumococcal conjugate vaccination on invasive pneumococcal disease and pneumonia in The Gambia: 10 years of population-based surveillance. Lancet Infect Dis [Internet]. 2021;21(9):1293–302. Available from: 10.1016/S1473-3099(20)30880-X. 10.1016/S1473-3099(20)30880-X PMC8384632 34280357

[16] WHO. Antimicrobial resistance. Global report on surveillance. World Heal Organ. 2014;61(3).

[17] Murray CJ, Ikuta KS, Sharara F, Swetschinski L, Robles Aguilar G, Gray A et al. Global burden of bacterial antimicrobial resistance in 2019: a systematic analysis. Lancet. 2022;399(10325). 10.1016/S0140-6736(21)02724-0 PMC8841637 35065702

[18] Ezzelle J, Rodriguez-Chavez IR, Darden JM, Stirewalt M, Kunwar N, Hitchcock R et al. Guidelines on good clinical laboratory practice: bridging operations between research and clinical research laboratories. J Pharm Biomed Anal. 2008; Vol. 46. 10.1016/j.jpba.2007.10.010 PMC2213906 18037599

[19] Mok D, Lim E, Bingham A. Identification of ISO 15189:2012 conformance requirements for medical laboratory internal auditing. Aust J Med Sci. 2015;36(1).

[20] International Organization for standardization. ISO 15189:2012 - Medical laboratories-Requirements for quality and competence. Int Organ Stand ISO. 2012;2012.

[21] Pai R, Gertz RE, Beall B. Sequential multiplex PCR approach for determining capsular serotypes of Streptococcus pneumoniae isolates. J Clin Microbiol. 2006;44(1). 10.1128/JCM.44.1.124-131.2006 PMC1351965 16390959

[22] Miller ML, Gao G, Pestina T, Persons D, Tuomanen E (2007). Hypersusceptibility to invasive pneumococcal infection in experimental sickle cell disease involves platelet-activating factor receptor. J Infect Dis.

[23] Battersby AJ, Knox-Macaulay HHM, Carrol ED. Susceptibility to invasive bacterial infections in children with sickle cell disease. Pediatr Blood Cancer Vol 55; 2010. 10.1002/pbc.22461 20232448

[24] Evans C, Orf K, Horvath E, Levin M, De La Fuente J, Chakravorty S et al. Impairment of neutrophil oxidative burst in children with sickle cell disease is associated with heme oxygenase-1. Haematologica. 2015;100(12). 10.3324/haematol.2015.128777 PMC4666326 26315932

[25] Adamkiewicz T, Thomas S, Tunali A, Lai K, Yee MM, Yildirim I (2021). Population-Based surveillance of pneumococcal infections in children with Sickle Cell Disease before and after Prevnar 7® and Prevnar 13® licensure: implications for expanded vaccination. Blood.

[26] Pitts SI, Apostolou A, Dasgupta S, Delgado N, Kirn TJ, Montana B et al. Serotype 10a in case patients with invasive pneumococcal disease: a pilot study of pcr-based serotyping in new jersey. Public Health Rep. 2015;130(1). 10.1177/003335491513000107 PMC4245285 25552755

[27] Pichichero ME, Khan MN, Xu Q. Next generation protein based Streptococcus pneumoniae vaccines. Volume 12. Human Vaccines and Immunotherapeutics; 2016. 10.1080/21645515.2015.1052198 PMC4962723 26539741

[28] Du Q, Shi W, Yu D, Yao K. hu. Epidemiology of non-vaccine serotypes of Streptococcus pneumoniae before and after universal administration of pneumococcal conjugate vaccines. Vol. 17, Human Vaccines and Immunotherapeutics. 2021. 10.1080/21645515.2021.1985353 PMC8903918 34726580

[29] Ansaldi F, De Florentis D, Canepa P, Bandettini R, Diana MC, Martini M et al. Epidemiological changes after PCV7 implementation in Italy: perspective for new vaccines. Vol. 7, Hum Vaccines. 2011. 10.4161/hv.7.0.14602 21546795

[30] Balsells E, Guillot L, Nair H, Kyaw MH. Serotype distribution of Streptococcus pneumoniae causing invasive disease in children in the post-PCV era: a systematic review and meta-analysis. PLoS ONE. 2017;12(5). 10.1371/journal.pone.0177113 PMC5423631 28486544

[31] Hanquet G, Krizova P, Dalby T, Ladhani SN, Nuorti JP, Danis K et al. Serotype replacement after introduction of 10-Valent and 13-Valent Pneumococcal Conjugate Vaccines in 10 countries, Europe. Emerg Infect Dis. 2022;28(1). 10.3201/eid2801.210734 PMC8714201 34932457

[32] Yun KW, Rhie K, Kang JH, Kim KH, Ahn JG, Kim YJ et al. Emergence of serotype 10A-ST11189 among pediatric invasive pneumococcal diseases, South Korea, 2014–2019. Vaccine. 2021;39(40). 10.1016/j.vaccine.2021.08.072 34465475

[33] Givon-Lavi 1Ben-ShimolS, Kotler N, van der Beek L, Greenberg BA, Dagan D (2021). Post-13-valent pneumococcal conjugate vaccine dynamics in young children of serotypes included in candidate extended-spectrum conjugate vaccines. Emerg Infect Dis.

[34] CDC CFDCAP. About pneumococcal vaccine. CDC- Centers for Disease Control and Prevention; 2017.

[35] Daw NC, Wilimas JA, Wang WC, Presbury GJ, Joyner RE, Harris SC et al. Nasopharyngeal carriage of penicillin-resistant streptococcus pneumoniae in children with sickle cell disease. Pediatrics. 1997;99(4). 10.1542/peds.99.4.e7 9099782

[36] Srisuwananukorn A, Han J, Raslan R, Gowhari M, Hussain F, Njoku F et al. Antimicrobial resistance is a risk factor for mortality in adults with sickle cell disease. Vol. 106, Haematologica. 2021. 10.3324/haematol.2020.267872 PMC8168496 33121239

[37] Donkor ES, Foster-Nyarko E, Enweronu-Laryea CC. Relationship between antibiotic resistance and sickle cell anemia: preliminary evidence from a pediatric carriage study in Ghana. Infect Drug Resist. 2013;6. 10.2147/IDR.S40062 PMC3733876 23930075

[38] Eliakim-Raz N, Lador A, Leibovici-Weissman Y, Elbaz M, Paul M, Leibovici L. Efficacy and safety of chloramphenicol: joining the revival of old antibiotics? Systematic review and meta-analysis of randomized controlled trials. J Antimicrob Chemother. 2014;70(4). 10.1093/jac/dku530 25583746

[39] Wiest DB, Cochran JB, Tecklenburg FW. Chloramphenicol Toxicity Revisited: a 12-Year-old patient with a brain abscess. J Pediatr Pharmacol Ther. 2012;17(2). 10.5863/1551-6776-17.2.182 PMC3470440 23118672

[40] Tshilolo L, Wembonyama S, Summa V, Avvisati G (2010). Hemogram findings in congolese children with sickle cell disease in remission. Med Trop (Mars).

[41] Omoti CE. Haematological values in sickle cell anaemia in steady state and during vaso-occlusive crisis in Benin City, Nigeria. Ann Afr Med. 2005;4(2).

[42] Iheanacho O. Haematological parameters of adult and paediatric subjects with sickle cell disease in steady state, in Benin City, Nigeria. Int Blood Res Rev. 2015;3(4).

[43] Nagose V, Rathod S. Hematological Profile of Sickle Cell Anemia subjects in Central India: A Cross Sectional Analysis. Ann Pathol Lab Med. 2018;5(1).

[44] Antwi-Boasiako C, Ekem I, Abdul-Rahman M, Sey F, Doku A, Dzudzor B et al. Hematological parameters in ghanaian sickle cell disease patients. J Blood Med. 2018;9. 10.2147/JBM.S169872 PMC6217132 30464671

[45] Augustina L, Alfred EF, Marcellinus NU. Some hematological and biochemical changes Associated with Blood Transfusion in Sickle Cell Anaemia Patients. J Mol Immunol. 2016;2(1).

[46] Ahmed SG, Ibrahim UA, Hassan AW. Hematological parameters in sick cell anemia patients with and without priapism. Ann Saudi Med. 2006;26(6). 10.5144/0256-4947.2006.439 PMC6074330 17143019

[47] Ahmed AE, Ali YZ, Al-Suliman AM, Albagshi JM, Salamah M, Al, Elsayid M et al. The prevalence of abnormal leukocyte count, and its predisposing factors, in patients with sickle cell disease in Saudi Arabia. J Blood Med. 2017;8. 10.2147/JBM.S148463 PMC5661844 29123434

[48] Wun T. The role of inflammation and leukocytes in the pathogenesis of sickle cell disease. Hematology. 2001;5(5). 10.1080/10245332.2000.11746536 27420932

[49] Allali S, Maciel TT, Hermine O, De Montalembert M. Innate immune cells, major protagonists of sickle cell disease pathophysiology. Haematologica; 2020. Vol. 105. 10.3324/haematol.2019.229989 PMC7012475 31919091

[50] Zhang D, Xu C, Manwani D, Frenette PS. Neutrophils, platelets, and inflammatory pathways at the nexus of sickle cell disease pathophysiology. Vol. 127, Blood. 2016. 10.1182/blood-2015-09-618538 PMC4760086 26758915

[51] Okpala I. The intriguing contribution of white blood cells to sickle cell disease - A red cell disorder. Vol. 18, Blood Rev. 2004. 10.1016/s0268-960x(03)00037-7 14684149

[52] Akinbami A, Dosunmu A, Adediran A, Oshinaike O, Adebola P, Arogundade O. Haematological values in homozygous sickle cell disease in steady state and haemoglobin phenotypes AA controls in Lagos, Nigeria. BMC Res Notes. 2012;5. 10.1186/1756-0500-5-396 PMC3423074 22849350

[53] Buchanan GR, Glader BE. Leukocyte Counts in Children with Sickle Cell Disease: comparative values in the steady state, Vaso-occlusive Crisis, and bacterial infection. Am J Dis Child. 1978;132(4). 10.1001/archpedi.1978.02120290068013 645659

[54] Clinical and Laboratory Standards Institute Performance standards for antimicrobial susceptibility testing. 2017. pp. M100–S27.

