# Supplementary material for: Laboratory indices of hospitalized sickle cell disease patients, prevalence and antimicrobial susceptibility of pathogenic bacterial isolates at MRCG ward in the Gambia
Source: BMC Infect Dis. 2023 Aug 22;23:546. doi: 10.1186/s12879-023-08542-z (PMC10464433; doi:10.1186/s12879-023-08542-z)
Supplement: Supplementary file 2 — Supplementary Material 2 [file 12879_2023_8542_MOESM2_ESM.docx]

| OPDNO | Age | Sex | HB | WBC | RBC | MCV | PLATS | HBGENOT | Pathogen Isolated |
| --- | --- | --- | --- | --- | --- | --- | --- | --- | --- |
| 2017/0182 | 0 | M | 9.1 | 12.9 | 3.67 | 68.6 | 247 | SS | Enterococcus species |
| 2021/4814 | 12 | F | 10.5 | 18.7 | 3.7 | 81.6 | 549 | SS | Salmonella species |
| 2018/6249 | 1 | F | 7.63 | 14 | 3.68 | 70.5 | 511 | SS | Salmonella species |
| 2016/4874 | 4 | M | 6.1 | 38.03 | 1.97 | 89.7 | 351 | SS | Shigella boydii |
| 2020/3271 | 3 | M | 8.7 | 30.1 | 3.74 | 68.5 | 426 | SC | Staphylococcus aureus |
| 2018/6451 | 1 | F | 7.13 | 25.7 | 2.51 | 99.7 | 257 | SS | Staphylococcus aureus |
| 2019/5440 | 0 | M | 8.8 | 15.44 | 3.06 | 87.2 | 861 | SS | Staphylococcus aureus |
| 2021/0956 | 6 | M | 10.5 | 9.36 | 4.66 | 63.5 | 245 | SC | Staph aureus |
| 2003/A804 | 32 | F | 10.4 | 8.49 | 3.02 | 101 | 512 | SS | Strep Pneumoniae (12F) |
| 2020/0167 | 0 | M | 8.8 | 13.01 | 3.77 | 74.2 | 511 | SS | Strep Pneumoniae (10A) |
| 2021/2426 | 1 | M | 6.7 | 17.88 | 2.93 | 69.4 | 786 | SS | Strep Pneumoniae (12F) |

Supplementary Table 1: Summary of pathogens isolated from sickle cell disease patients

This table showed the different types of pathogens isolated from blood cultures of sickle cell disease patients and their haematological laboratory indices. 12F and 10A are different *Streptococcus Pneumonia*e serotypes. OPDNO: Outpatient Department Number; HB: Haemoglobin; WBC: White Blood Cells; RBC: Red Blood Cells; MCV: Mean Cell Volume; PLATS: Platelets; HBGENOT: Haemoglobin Genotype
